# The Science of Colour in Rice: A Review on Health Benefits, Genetic Regulation, and Anthocyanin Stability

**DOI:** 10.1186/s12284-026-00911-x

**Published:** 2026-06-05

**Authors:** Farhat Jahan, Priyadarsini Sanghamitra, Durga Prasad Barik, Jitendriya Meher, Manoj Patra, Nabaneeta Basak, Gaurav Kumar, Sutapa Sarkar, Reshmi Raj KR, Sushanta Kumar Dash, Kambaska Kumar Behera

**Affiliations:** 1 Department of Botany and Biotechnology, Ravenshaw University, Cuttack, Odisha India; 2 ICAR Central Rice Research Institute, Cuttack, Odisha 753006 India; 3 Department of Pharmacognosy, AIPER, Cuttack, Odisha 754022 India

**Keywords:** Pigmented rice, Anthocyanins, Flavonoids, Proanthocyanidins, Pigmentation, Genetic regulation, Antioxidant properties, Health benefits, Anthocyanin stability, Synthesis, Degradation, Bioactive compounds

## Abstract

The pigmented rice types, i.e., black, red, and purple rice, are highly nutritious substitutes for the ordinary white rice, providing compounds such as anthocyanins, flavonoids, and proanthocyanidins. These bioactive compounds confer antioxidant, anti-inflammatory, anti-cancer, anti-diabetic, and cardiovascular-protective effects, while also assisting in weight management, gut health, and metabolism. The genetic basis of pigmentation includes regulatory genes like OsC1, OsDFR, and OsMYB, which comprise the MYB-bHLH-WD40 complex regulating anthocyanin biosynthesis. Anthocyanin pathways interact with flavonoid and proanthocyanidin synthesis, which is essential for colouration and stress adaptation. The environmental factors, such as temperature, pH, and storage conditions, cause degradation of anthocyanins. Acylation and encapsulation are techniques used in preserving anthocyanins better for industrial use. However, degradation of anthocyanins remains unexplored. Emerging evidence shows that degradation is not only chemical but also enzymatically regulated, mediated by polyphenol oxidases, peroxidases, and β-glucosidases, which accelerate oxidation and hydrolysis reactions and contribute to organ-specific pigment loss. Studies confirm first-order kinetics of anthocyanin breakdown under heat, alkaline pH, and oxygen, while stability improves with encapsulation, co-pigmentation, and gamma irradiation. This review explores the pigmentation of rice (*Oryza sativa* L.), focusing on its respective health benefits, genetics, synthesis, and degradation. Additionally, much remains to be discovered about its genetic and molecular basis. The regulation of vacuolar enzymes and degradation-related genes in rice tissues is still poorly understood, representing a major knowledge gap compared with biosynthesis.

## Introduction

**Fig. 1:**
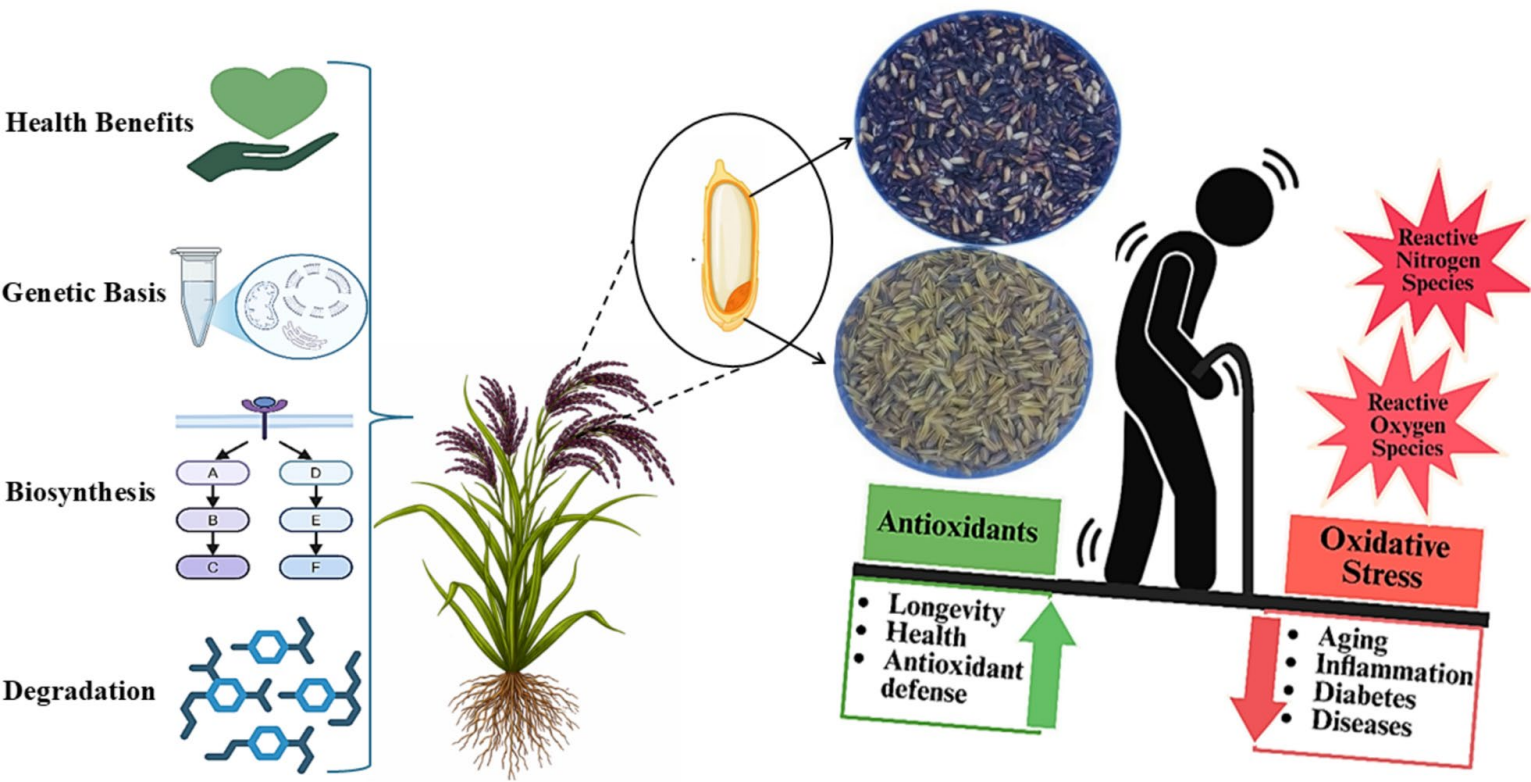
Schematic Abstract

Rice, wheat, and maize constituting 90% of all cereal crops provide carbohydrates, proteins, vitamins, minerals, and oils indispensable for health and food security (Lap et al. 2021). Thus, rice remains as a major cereal crop and the staple food of more than half of the worldwide population. It is cultivated in almost every region, including 17 nations across Asia and the Pacific, 9 American countries, and 8 African nations except Antarctica. India alone has nearly 6,000 varieties (Kumar et al. 2020). Its cultivation is vital in South and Southeast Asia. However, the demand for rice is still rising, especially in Asia, where China and India produce most of the world's rice (more than 90%). India produces 165.3 million tons of rice per year on 44.1 million hectares of cultivation. This indicates the need for rice breeding and cultivation improvements to enhance productivity and yield stability (Manoj et al. 2023; Kesh and Kaushik 2020). Therefore, meeting the future demand for rice is crucial for food and nutrition security. In many countries, people eat polished rice grains, known as white rice, and the remaining outer part of brown rice, known as bran. Milling removes most of the nutrients from the outer layers and embryo of rice, leaving only starch in the inner part (Mbanjo et al. 2020; Bhat et al. 2020) (Fig. 1).

The Traditional rice landraces were richer in bioactive compounds and had higher nutritional value than modern varieties (Mbanjo et al. 2020). Conversely, there is a rise in incidence of non-communicable diseases which includes cardiovascular diseases, Type II diabetes, obesity, and cancer in both developed and developing countries. In such conditions, over dependence on white rice can lead to nutrient deficiencies (Wongsa 2020). Epidemiological studies show that the low occurrence of some chronic diseases in rice consuming regions might be connected to the high level of antioxidants present in rice (Goufo and Trindade 2014). Hence, there is a growing interest in healthier diets, leading to a demand for nutritious rice that will benefit both consumers and producers (Mbanjo et al. 2020).

Besides white rice, nutritionally superior pigmented rice are characterized by the accumulation of anthocyanins and proanthocyanidins in the pericarp and aleurone layers (Kumar et al. 2020). However, Brown rice is unpolished white rice and not genetically pigmented, while yellow rice colouration is rare, carotenoid-based, and not conventionally grouped under pigmented rice (Idrishi et al. 2024; Mbanjo et al. 2020; Mackon et al. 2021). Pigmented rice can be incorporated into many kinds of diets, which can be beneficial in addressing various lifestyle and nutrient-deficient diseases because they are rich in anthocyanins, proanthocyanidins, and flavonoids. Because of their nutritional, functional, and digestibility significance, these varieties of rice are now widely studied for their health benefits, nutritional composition, phytochemicals, cooking properties, value addition, glycaemic values, and bioactivity (Sudan et al. 2023).

## Overview of Pigmented Rice

### Pigmentation in Rice

**Fig. 2:**
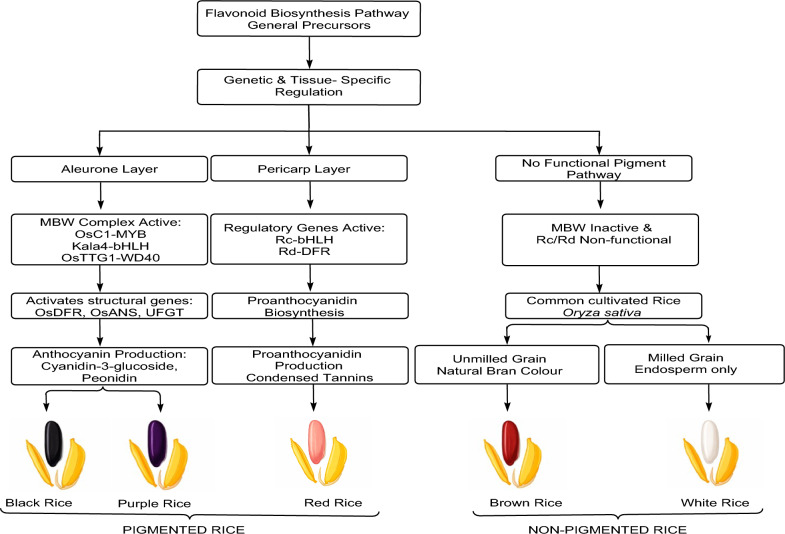
Major types of pigmented rice varieties

Pigmented rice varieties are classified as black, purple and red rice (Idrishi et al. 2024). In contrast, anthocyanins are the major pigments responsible for black and purple rice colours whereas the colouration of red rice is due to proanthocyanidins, which are also called condensed tannins (Mbanjo et al. 2020; Idrishi et al. 2024) as indicated in Fig. 2. In addition, other polyphenols, such as catechins and flavanols, are responsible for red rice being more intense and stable (Shao et al. 2018; Mackon et al. 2021). Still, carotenoids are almost negligible and cause very less yellow colourization of rice. Therefore, they do not rank among the major pigments truly responsible for colouration in rice (Mbanjo et al. 2020; Mackon et al. 2021).

The intensity of the variation in pigmentation confirms that polygenes control the characteristics but there are still several unidentified genes (Ham et al. 2015). Pigmented varieties of rice contain high levels of polyphenols (ranging from 250 to 1075 mg/100 g) as well as high antioxidant activity (up to 69.93% DPPH inhibition) (Krishnan et al. 2020; Pradipta et al. 2023). Thus, the main constituents including anthocyanins, proanthocyanidins, flavonoids, and phenolic acids are mainly located in the pericarp and aleurone layers. Besides, pigmented rice has also been found to be richer in Fe, Zn, and Mn but poorer in P when compared with white rice (Shao et al. 2018). Black and purple rice is however richer in Fe and Zn amount along with Ca, Cu, and Mg as compared to red rice (Shao et al. 2018; Gogoi et al. 2024). It has been recently indicated that the mineral accumulation is a phenotypic trait controlled by genetics and not necessarily linked to the presence of anthocyanins (Gogoi et al. 2024). Altogether, the mentioned traits make pigmented rice a highly nutritious functional cereal that significantly contributes to the antioxidant and nutraceutical areas.

### Global Distribution of Pigmented Rice

The Nutritional content, methods of cooking, and uses of rice vary depending on its size, shape, and colour. The two leading producers of rice worldwide are China and India, accompanied by Indonesia, Bangladesh, Vietnam, Myanmar, and Thailand (Krishnan et al. 2020). Almost all over the Asian subcontinent, pigmented purple rice has been grown every year. However, all varieties of Pigmented rice are gaining popularity for their production. Countries like China, India, Japan, Vietnam, Sri Lanka, Thailand, Indonesia, Korea, Laos, and Nepal, as well as Brazil and a few other nations have been listed as major producers of pigmented rice varieties till date. On the other hand, countries like Australia, Italy, France, and Russia have all introduced new coloured rice varieties onto the international market. There are several varieties of black or purple rice, such as Japanese black rice, Italian black rice, black sticky/glutinous rice, and Thai black jasmine rice. India is a leading producer, with major states producing pigmented rice varieties across India (Sharma et al. 2024).

### Evolutionary History

Pigmented rice variety has an old history interlinking the rice domestication in South and Southeast Asia. According to the archaeological findings from Lahuradewa located in the Middle Ganges plains, rice cultivation was practiced as early as 6409 BC, thus making India to be one of the main centres for early rice cultivation (Tewari et al. 2008). Since the Indus Valley civilization (3000–1500 BC) diverse landraces, including pigmented forms were cultivated within the traditional agro-ecological systems; during this period the domestication of rice became widespread across the Indian subcontinent (Sharma et al. 2024). Before the Green Revolution, India had a very rich diversity of rice with a multitude of pigmented landraces. In sharp contrast, the widespread adoption of high-yielding white rice varieties led to the decline of these traditional, nutritionally rich forms (The Hindu Group 2012; Devi et al. 2015).

However, the evolutionary history states that, pigmented rice was regarded as an adaptive trait that came into existence because of environmental stresses and the subsequent selection of the trait by humans during the domestication process. The pigments found in black, purple and red rice, specifically anthocyanins and proanthocyanidins, guard the plants through protection against high light intensity, temperature extremes, and oxidative stress, which in return increases the survival rate of the plants in the stress-prone environments (Lee 2010). In addition, the mutations in the regulatory genes such as Kala4 resulted in the enhancement of anthocyanin accumulation which in turn contributed to the emergence and spread of black rice phenotypes (Oikawa et al. 2015; Kumane and Singh 2016). Apart from this, the population-genomic studies that were conducted on a total of 5104 global rice accessions, out of which 2794 were pigmented, revealed that the cultivated red rice was descended from the wild rice and diverged from the white rice about 3500 years ago, with frequent shifts between wild, cultivated, and weedy forms. The wild rice known as *Oryza rufipogon* serves as the direct ancestor of cultivated rice while showing a constant red pericarp colour through its proanthocyanin content. Red rice developed from this trait which remained in use during the first phase of rice domestication between 6000 and 2000 B.C. Red rice originated from wild populations according to genomic data which showed that it maintained better environmental adaptability and stress tolerance than white rice. However, human breeding practices selected specific mutations which led to the emergence of white rice because they caused the Rc gene to lose its function. Red rice served as a genetic link which permitted weedy rice to develop from domesticated rice and then return through re-domestication while keeping essential features that evolved during rice domestication. On top of that, genome-wide association studies led to the identification of new candidate genes that are linked to red-pericarp pigmentation, thus providing solid genomic support for the evolutionary origin and adaptive survival of pigmented rice (Xi et al. 2024).

### Role of Pigments in Plant-Environment Interactions

Anthocyanins are the mediators of plant-environment interactions, as they integrate various signals of the environment with the molecular and physiological responses of the plants. The most important environmental factor is the light which mainly regulates anthocyanin accumulation, and through the activation of the main structural genes of the biosynthetic pathway (such as CHS, DFR, ANS, and UFGT) by photoreceptors like phytochromes and cryptochromes (Do et al. 2023). Moreover, light quality and intensity have a combined effect on temperature, where low temperatures support anthocyanin production, while high temperatures inhibit pigment accumulation by the downregulation of pathway genes (Do et al. 2023).

Along with other plant responses to environmental stresses, drought and UV radiation which are further intensify the accumulation of anthocyanins and flavonoids—secondary metabolites that play a major role in plant stress tolerance due to their ability to effectively scavenge reactive oxygen species (ROS) and to absorb UV rays (Jan et al. 2022). Accumulation of the non-pigment antioxidant flavonoids even to the point of overaccumulation plays a keyrole in maintaining the stability of photosynthesis and improving the tolerance of rice plants to the combined drought and UV stress (Jan et al. 2022). The availability of nutrients in the soil, especially the carbon and nitrogen status, also influences the process of anthocyanin synthesis by changing the expression of the genes in the phenylpropanoid pathway and interaction in signalling mediated by salicylic acid (Jan et al. 2020). In the same vein, transcriptomic data show the connection of anthocyanin-mediated stress response to the hormone interaction involving abscisic acid and jasmonic acid, thus facilitating the coordinated crosstalk of the abiotic and the biotic stress responses (Jan et al. 2020; Kim et al. 2018; Ahmadzai et al. 2025). Therefore, it can be concluded that plant tissues rich in anthocyanins regulates hormonal signalling, improves UV resistance, and strengthens the plant against biotic and abiotic stresses as well as protect the leaf tissues from oxidation and photodamage keeping the photosynthetic efficiency throughout the process (Manoj et al. 2023).

## Health Impacts: Antioxidant Properties, and Nutritional Balance

Pigmented rice is more nutritious and contains antioxidant nutraceuticals, which are beneficial in regulating metabolism impairment and supporting human health as listed in Table 1, Health benefits of pigmented rice. Anthocyanins and proanthocyanins present in pigmented rice have anti-cancer, anti-allergic, anti-aging, anti-diabetic, and anti-obesity properties and help to treat major diseases like ulcers, fractures, burns, and skin lesions (Kumar et al. 2020).

**Table 1:** Health benefits of pigmented rice

Sl. no.	Bioactivity	Research level	Sample characteristics	Core evidence/health benefits	References
1	Antioxidant activity	Cellular (In vitro)	Chemical antioxidant assays; cultured cell systems	Flavonoids, phenolic compounds, tocopherols, and anthocyanins scavenge ROS and maintain cellular redox balance	Ghasemzadeh et al. (2018)
		Animal (In vivo)	Rodent oxidative stress models	Reduction of oxidative stress and inflammation-associated damage	Krishnanunni et al. (2014); Zhang et al. (2015); Zhang et al. 2018
		Human (Epidemiological)	Population-based dietary studies	Consumption associated with reduced risk of hyperglycemia, hypertension, cardiovascular diseases, neurodegenerative disorders, cancer, and aging	Kumar et al. (2020); Wongsa (2020)
2	Anti-diabetic activity	Cellular (In vitro)	Enzyme inhibition assays	Inhibition of α-amylase and α-glucosidase, delaying carbohydrate digestion	Wahyuni et al. (2016)
		Animal (In vivo)	Diabetic and high-fat diet rodent models	Improved glucose metabolism and insulin sensitivity; γ-oryzanol alleviates hypothalamic ER stress	Boue et al. (2016); Chiou et al. (2018); Chandramouli et al. (2017)
		Human (Epidemiological/Intervention)	Dietary intervention and cohort studies	Reduced glycemic index; improved postprandial glucose response; protection against type-2 diabetes compared with white rice	Chen et al., (2016); Reddy (2018); Devi and Badwaik (2022)
3	Hypertension	Human (Epidemiological)	Nutrient-intake population studies	Magnesium in red and brown rice regulates sodium balance and maintains normal blood pressure	Kumar et al. (2020); Rathna Priya et al. (2019)
4	Anti-obesity effect	Animal (In vivo)	Rodent diet-induced obesity models	Downregulation of lipid absorption genes (NPC1L1, ACAT2, MTTP); prebiotics enhance gut microbiota	Krishnan et al. (2020)
		Human (Epidemiological)	Dietary pattern analysis	High fiber and antioxidant content reduces weight gain, inflammation, and improves gut health	Kumar et al. (2020); Rathna Priya et al. (2019)
5	Anti-cancer activity	Cellular (In vitro)	Breast, liver, and leukemic cancer cell lines	Rice extracts and phytosterols (β-sitosterol, stigmasterol) inhibit cancer cell proliferation	Ghasemzadeh et al. (2018); Chen et al. (2016); Devi and Badwaik (2022)
		Animal (In vivo)	Rodent cancer models	Anti-mutagenic and tumor-suppressive effects of bran bioactives	Devi and Badwaik (2022)
		Human (Epidemiological)	Population dietary studies	Reduced cancer risk linked to fiber, selenium, manganese, anthocyanins, and proanthocyanidins	Kumar et al. 2020; Rathna Priya et al. 2019
6	Anti-inflammatory properties	Cellular & Animal	Cell-based inflammation assays; rodent models	Phenolics, bioflavonoids, anthocyanins reduce inflammatory mediators	Riar and Bhat (2015); Siroha et al. (2023)
		Human (Epidemiological)	Population studies	Lower incidence of inflammation-driven diseases such as CVD and cancer	Kumar et al. (2020); Wongsa (2020)
7	Medicinal properties (Traditional)	Ethnomedicinal	Ayurvedic texts and regional folk practices	Therapeutic use for fever, ulcers, wounds, gastrointestinal disorders, skin diseases, blood pressure, and postpartum care	Kumar et al. (2020); Bhat et al. (2020); Rathna Priya et al. (2019)
8	Skin anti-aging properties	Cellular (In vitro)	Skin and cancer cell line assays	Procyanidins and flavan-3-ols exhibit anti-aging and anti-metastatic activity (HER2/ErbB2 pathway)	Luo et al. (2014)
		Applied/Industrial	Nutraceutical and cosmetic formulations	Bio-actives (catechin, vanillic acid, oryzanol) incorporated into anti-aging products	Kumar et al. (2020)
9	Anti-allergic effect	Human (Clinical observation)	Dietary intolerance and allergy management	Hypoallergenic rice protein; gluten-free suitability for celiac patients	Kumar et al. (2020); Rathna Priya et al. (2019)
10	Anti neurodegenerative potential	Cellular (In vitro)	SH-SY5Y neuronal cell models	Protection against H₂O₂-induced oxidative neuronal damage	Vargas et al. (2018)
		Human (Epidemiological inference)	Dietary flavonoid intake studies	Lower risk of Alzheimer’s and Parkinson’s diseases	Wongsa (2020)
11	Cardiovascular disease treatment	Animal (In vivo)	High-fat diet rat models	Reduced cardiovascular risk markers with anthocyanin-rich black rice	Shao and Bao (2015)
		Human (Epidemiological)	Population nutrition studies	Reduced metabolic syndrome and heart attack risk linked to brown and red rice intake	Wongsa (2020); Rathna Priya et al. (2019); Vargas et al. (2018)
12	Cholesterol management	Animal & Human	Rodent models; dietary intervention studies	Lower LDL cholesterol and reduced atherosclerotic plaque formation	Chen et al. (2016); Rathna Priya et al. (2019)

## Genetic Basis of Pigmentation in Rice

### Genetic Regulatory Mechanism of Anthocyanin Biosynthesis

The biosynthesis of rice anthocyanin is regulated by the late-pathway structural genes and the transcriptional factors that determine the specific pigmentation of the tissues involved, such as leaves, hulls, pericarps, apiculi, and stigmas. OsDFR (dihydroflavonol reductase) and OsANS (anthocyanidin synthase) are examples of structural genes which has been listed in Table 2, that act on the last steps of flavonoid pathway, but their expression depends on the MYB-bHLH-WD40 (MBW) transcriptional complex, which is conserved across species and is required for anthocyanin production in rice (Petroni and Tonelli 2011; Xu et al. 2015; Xia et al. 2021; Mackon et al. 2021; Meng et al. 2021). R2R3-MYB transcription factors, mainly OsC1 and related members like Kala3 and OsPL (Purple Leaf), play a significant role in the MBW complex by providing promoter specificity through their interaction with cis-regulatory elements (e.g., AC-box motifs) of anthocyanin production genes (ElShamey et al. 2025). The natural variation and functional loss mutations in OsC1 are strongly linked to the breeding that has resulted in the loss of pigmentation traits like purple apiculus, stigma, and leaf sheath colour (Xia et al. 2021; Zheng et al. 2019; Haghi et al. 2022).

**Table 2:** Components of the MYB-bHLH-WD40 (MBW) Complex Regulating Anthocyanin Biosynthesis in Rice

Sl. no.	Component	Identified genes	Interaction mode	Major target genes	Regulatory role & phenotypic impact	References
1	MYB	OsC1, Kala3, OsPL	DNA binding; MYB-bHLH interaction	OsDFR, OsANS	Master regulators of pigmentation; domestication-associated loss of colour	Xia et al. (2021); Zheng et al. (2019)
2	bHLH	OsB2 (Kala4), OsB1/OsRb, Rc	Heterodimerization with MYB	OsDFR, OsANS	Tissue specificity; pericarp pigmentation via promoter rearrangement	Oikawa et al. (2015); Mackon et al. (2021); Yang et al. (2021)
3	WD40	OsTTG1 / OsPAC1	Scaffold for MYB-bHLH	MBW complex	Enhances transcriptional efficiency; loss abolishes pigmentation	Meng et al. (2021); Zheng et al. (2019)
4	Structural genes	OsDFR, OsANS, CHS, F3H	Activated by MBW	Anthocyanin pigments	Execute pigment biosynthesis	Xia et al. (2021); Mackon et al. (2021)

The bHLH transcription factors, such as OsB2 (Kala4) and OsB5/OsRb, are in complex with MYB proteins and for generating tissue-specific activation of anthocyanin biosynthesis. One significant event in the process of conservation and cultivation was moving the promoter around in Kala4, which then made the gene produce a lot of colourations in the non-edible part (pericarp) and led to the black rice grains containing anthocyanins (Oikawa et al. 2015; Mackon et al. 2021). On the other hand, the bHLH factor Rc still being a co-dependent control the proanthocyanidin branch in the pericarp (Yang et al. 2021). The WD40 protein OsTTG1 (OsPAC1) brings together the MBW complex and the transcriptional output is thereby increased. The OsTTG1 knockout done using CRISPR/Cas9 leads to a huge reduction in anthocyanin in different tissues, which then confirms that it is a key regulator (Meng et al. 2021; Yang et al. 2021). The interactions between proteins revealed direct contact among OsTTG1, OsC1, and Kala4, thus placing the WD40 proteins right in the middle of the rice anthocyanin regulatory network (Zheng et al. 2019; Yang et al. 2021).

The regulation of anthocyanin in rice is influenced not only by the above mentioned factors but also by the environment and the evolution. It is reported that light is an inducer while high temperature is a suppressor of the OsTTG1 expression, thus linking the external factor of temperature to pigment production (Yang et al. 2021; ElShamey et al. 2025). Through transcriptomic and co-expression network analyses, auxiliary regulators have been identified, one of them being OsWRKY57 in *Arabidopsis* ortholog (TTG2) is involved in the regulation of pigments (Yang et al. 2021). Population genetic studies indicate that there is very little functional redundancy among the WD40 genes and that there is directional selection on the OsTTG1 haplotypes between the indica and japonica subspecies, which again highlights the domestication-associated regulatory divergence (Yang et al. 2021).

Therefore, the genes OsC1 (MYB), OsRb/Rc/Kala4 (bHLH), and OsTTG1 (WD40) together create the main regulatory network that controls anthocyanin production in rice. They regulate this by activating the production of OsDFR and OsANS gene expression through the MBW complex (Zhao et al. 2016; Zheng et al. 2019; Xu et al. 2021). New findings suggest the existence of a rice-specific hierarchy that bHLH factors (such as S1/Kala4) to OsC1, which then causes the expression of the WD40 gene to be activated, thus putting the rice regulatory model apart from that of *Arabidopsis* (Sun et al. 2022). This combined MBW network not only allows for the precise control of the location, environment, and rice varieties in which anthocyanin is to be produced, but also for their responsiveness to factors such as space and environment.

### Genetic Basis

Genes identified through genetic means and resequencing as major candidates include Os06g0205100, a MYB-type gene very much involved in pigmentation regulation, whereas Os06g0193400 (bHLH) acts in phosphate starvation responses and possibly pigmentation (Haghi et al. 2022). The MBW complex controls tissue-specific pigmentation. For example, the OsC1-MYB protein interacts with either one of the two bHLH factors, OsPa or OsPs, to control pigmentation in apiculi and stigmas (Meng et al. 2021).

### Gene Duplication, Diversification, and Evolution

Duplication and diversification of genes have been studied in numerous rice pigmentation genes. Primary regulatory genes of pigment biosynthesis, such as OsC1 and OsB2 (bHLH) exist in several allelic forms affecting pigment accumulation by way of evolutionary adaptation (Xia et al. 2021). The gene clusters, OsPa, OsPs, and S1, mapped to chromosome 4, are structurally similar yet diversified in function for tissue-specific pigmentation (Meng et al. 2021). Duplication of pigmentation-related genes like OsMYB and bHLH has led to the functional evolution and wide phenotypic expression of pigmentation in rice. Rearrangements in the Kala4 promoter have been associated with ectopic pericarp expression and, in turn, black rice pigmentation (Mackon et al. 2021). Structural genes such as chalcone synthase (CHS), chalcone isomerase (CHI), and flavonoid hydroxylases (F3′H, F3′5′H) have functions in anthocyanin biosynthesis activities, contributing later to duplication and diversification. Allelic variants of these genes (e.g., F3′H) differentiate wild and cultivated rice. SNPs located on chromosomes 2, 4, and 6 have been detected by the GWAS approach to point to the presence of WD40 and bHLH transcription factors involved in pigmentation regulation in rice (Haghi et al. 2022).

## Biosynthesis of Anthocyanins and Proanthocyanidins in Pigmented Rice

### Anthocyanin Biosynthesis Pathway

**Fig. 3:**
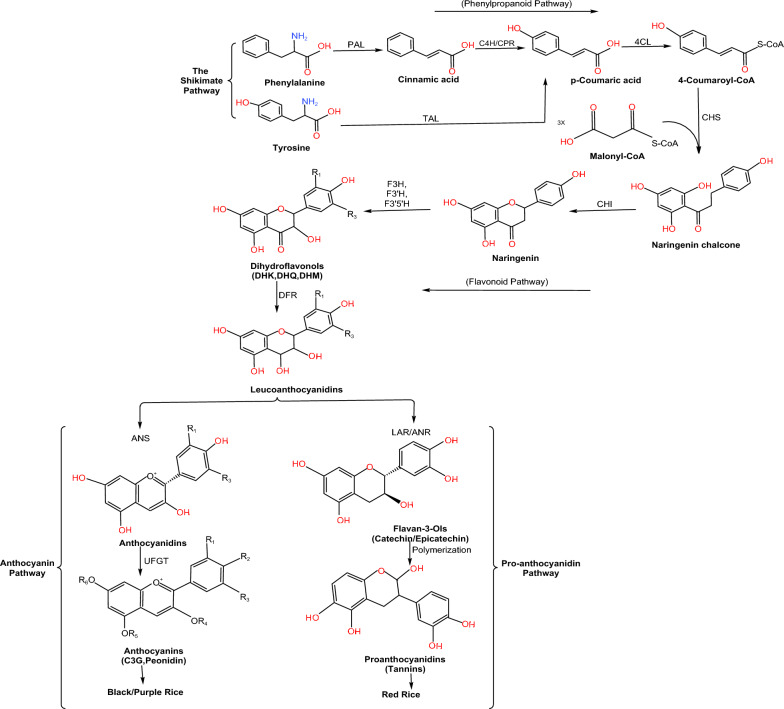
Anthocyanin and Pro-anthocyanidin Biosynthesis Pathway

As depicted in Fig. 3. Anthocyanin Biosynthesis Pathway shows that, Anthocyanins and their derivatives, such as flavonoids and proanthocyanidins, are produced by a branch of the general flavonoid pathway, with specific enzymes such as CHS (chalcone synthase), CHI (chalcone isomerase), DFR, and ANS (anthocyanidin synthase) involved (Xia et al. 2021). However, pigment production within the tissues depends upon the gene expression pattern regulated in a tissue-specific manner, with OsDFR favouring anthocyanin synthesis. During anthocyanin biosynthesis, gene products carrying the structural functions are CHS, CHI, F3H, F3'H, ANS, and UFGT, while their activity is controlled by transcription factors R2R3-MYB, bHLH, and WD40 among others (Meng et al. 2021).

The entire pathway of anthocyanin formation begins with the amino acid phenylalanine, which is finally transformed into 4-coumaroyl-CoA by means of the enzymes PAL, C4H, and 4CL. Subsequently, CHS steps in and utilizes 4-coumaroyl-CoA and malonyl-CoA to create naringenin chalcone. The enzyme chalcone isomerase (CHI) then changes this naringenin chalcone to naringenin. Flavonoid hydroxylases (F3′H, F3′5′H) then perform a hydroxylation reaction on naringenin resulting in the formation of dihydroflavonol. OsDFR reduces dihydroflavonol to leucoanthocyanidins. Leucoanthocyanidins are further converted into anthocyanidins by anthocyanidin synthase (ANS). The last conversion involves the glycosylation of the anthocyanidins by UFGTs, rendering stabilized anthocyanins that are stored in vacuoles (Mackon et al. 2021; Haghi et al. 2022).

### Proanthocyanidin Biosynthesis Pathway

Proanthocyanidins are condensed tannins because they exist as flavan-3-ol compounds that form both oligomeric and polymeric structures which provide plant physiological functions alongside human health advantages. The pathway starts from basic flavonoids which produce flavan-3-ol monomers that include catechin and epicatechin. Multiple structural and regulatory genes yet they remain unable to identify all the biochemical pathways which control PA polymerization and the methods which determine polymer structure (Constabel 2018). The Rc gene in rice controls PA accumulation in the pericarp because it functions as a bHLH transcription factor which regulates essential structural genes such as OsDFR. PA biosynthesis together with anthocyanin production uses phenylpropanoid-derived precursors but the two processes break apart during the leucoanthocyanidin stage when leucoanthocyanidin reductase and anthocyanidin reductase direct metabolites toward PA production (Meng et al. 2021; Mackon et al. 2021).

The structural makeup of PAs consists of flavan-3-ol units which interconnect through C4-C8 and C4-C6 interflavan bonds, while their polymerization range beginning from small oligomers with a degree of polymerization between 2 and 3 extends to highly polymerized structures which exceed a degree of polymerization of 20. The stability and biological activity of PA compounds experience changes because their DP level shows strong dependency on their natural distribution pattern (Fischer et al. 2014; Dixon and Sarnala 2020). The process of glycosylation takes place during the monomeric flavonoid intermediate stage, which leads to better solubility and transport ability, while mature PAs exist without glycosylation because their carbon–carbon interflavan linkages remain intact. The structural diversity of PA compounds develops through three main factors, which include monomer composition and linkage type and degree of polymerization (Haghi et al. 2022; Mackon et al. 2021; Fischer et al. 2014; Dixon and Sarnala 2020).

Metabolomic research shows that controlling upstream flavonoid pathways results in increased production of PA because external trilobatin application raised both oligomeric PA and total flavonoid content in purple rice grains without altering their polymer structure (Xiong et al. 2024). The main control mechanism of PA biosynthesis transcriptional regulation operates through MYB transcription factors, but scientists have not yet discovered the genes and enzymes which define PA polymer length and structural details (Constabel 2018).

### Epigenetic Modifications in Anthocyanin Regulation

Epigenetic mechanisms, such as DNA methylation and histone modifications, affect pigmentation-related genes in rice. These types of epigenetic modifications dictate the expression of genes and the accumulation of pigments in determined tissues (Xia et al. 2021). Here exists a correlation between methylation patterns in gene promoters like OsC1, OsDFR, OsPa, and OsPs and the expression of pigment intensity in different tissues. On the contrary, there are cases wherein promoter and coding sequence variations found in rice accessions could not be associated directly with any epigenetic regulation of pigmentation anywhere (Meng et al. 2021; Haghi et al. 2022). Setting of anthocyanin pathway genes by histone modification affects the expression of these genes and, in turn, controls anthocyanin accumulation. Recent studies have provided support for the involvement of such epigenetic factors in colour modulation (Mackon et al. 2021; Haghi et al. 2022). Mutations in regulatory genes responsible for pigmentation bring distinct colourations by missing out synthesis of anthocyanins. Mutations of OsC1 and OsB2 cause colourless phenotypes due to impaired regulation of anthocyanin biosynthesis (Xia et al. 2021). Loss-of-function mutations in OsDFR and Kala4 abolish pigmentation, with nucleotide variations in OsDFR causing premature stop codons and non-pigmented rice (Mackon et al. 2021). Single-nucleotide polymorphisms in OsC1, meaning a change in anthocyanin production. Mutants for activating components of the MBW complex, therefore, tend not to accumulate the pigment effectively (Haghi et al. 2022).

## Anthocyanin Transport and Storage

**Fig. 4:**
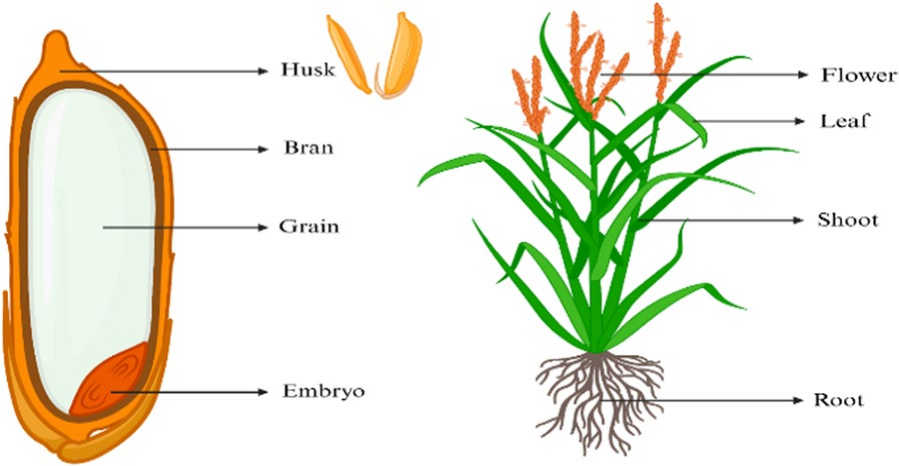
Different Parts of *O. sativa*

Table 3 explains how anthocyanins are accumulated and controlled in pigmented rice. It lists the genes and factors that produce pigments in parts like the hull, pericarp, and bran. The table also shows how pigments are distributed differently in each plant part depicted in Fig. 4.

**Table 3:** Anthocyanin Synthesis and Regulation Across Different Parts of the Pigmented Rice

Sl. no.	Organs of pigmented rice	Anthocyanin regulation and pigmentation	Major genes/factors involved	References
1	Hull	Hull pigmentation is controlled by the gene system C-S-A. The C1 (MYB transcription factor) works in conjunction with S1 (bHLH protein) and A1 (DFR) to bring about anthocyanin synthesis and purple colouration of the hulls	C1 (MYB), S1 (bHLH), A1 (DFR)	Galland et al. (2014); Sun et al. (2018)
2	Pericarp	Kala4 and OsC1 regulate the accumulation of anthocyanins, which results in the black pericarp phenotype. Functional alleles in these genes allow anthocyanin accumulation, whereas null mutations result in unpigmentation. Rc interacts with OsDFR to regulate biosynthesis of proanthocyanidins, a pigment that colours. Anthocyanin mainly accumulates in the pericarp and aleurone layers, giving black rice its trademark dark colour. Enzymes like F3'H serve in the production of anthocyanins or proanthocyanidins, depending on cultivar genetics. Kala4 and OsC1 act functionally to produce pigmentation. Localization of anthocyanin occurs in aleurone layers with a blackness effect. Synthesized through a flavonoid pathway. CHS, CHI, and F3H convert dihydroflavonol that gets converted into leucoanthocyanidins by DFR. ANS converts leucoanthocyanidins into anthocyanidins that get stabilized by UGT Proanthocyanidins come from leucoanthocyanidins via the LAR, producing catechin monomers that polymerize	Kala4, OsC1, Rc, OsDFR CHS, CHI, F3H, DFR, ANS, UGT LAR, Rc, Rd	Meng et al. (2021); Haghi et al. (2022); Galland et al. (2014); Sun et al. (2018)
3	Seed Coat	Anthocyanins are present in the seed coat, contributing to the pigmentation of the rice grain	–	Meng et al. (2021)
4	Testa	Co-localization of pigments provides protective roles Proanthocyanidins are linked to seed dormancy in some varieties	Rc, Rd, CHS, CHI, F3H	Galland et al. (2014)
5	Embryo	High levels of flavones, including O- and C-glycosylated forms, act as protective agents. Most flavonoids are localized in the embryo coleoptile	CHS, CHI, F3H, OsFNS, CYP93G2	Galland et al. (2014)
6	Bran	97% of anthocyanins in rice are localized in the bran, which encompasses the pericarp, aleurone, and seed coat. Minimal amounts of anthocyanins are found in the endosperm	Rc, Rd	Meng et al. (2021); Galland et al. (2014)
7	Leaf	OsB2 (Kala 4) and OsRb regulate leaf anthocyanin and proanthocyanidin accumulation, with tissue-specific expression patterns	OsB2 (Kala4), OsRb	Meng et al. 2021
8	Stem and Leaf Sheath	Anthocyanin synthesis in the sheath is controlled by OsC1, wherein allelic variations influence the accretion level. Accumulation of anthocyanin occurs in the outer leaf sheaths (purple stem). There are major genes such as OsC1 and bHLH factors. The pigmentation initiates on the outer leaf sheath. They are controlled by OsC1, bHLH, and other transcription factors. GWAS identified SNPs associated with pigmentation on chromosomes 2, 4, and 6	OsC1 bHLH	Xia et al. (2021); Haghi et al. (2022); Galland et al. (2014)
9	Stigma	From the stigma, purple pigmentation is affected by OsC1 and Rb. OsC1 controls the amount of cyanidin 3-O-glucoside, and mutations in the genes could also result in decreased anthocyanin accumulation. OsC1, OsDFR, OsPa, and OsPs act in anthocyanin biosynthesis in stigmas and thus determine pigmentation in stigmas	OsC1, Rb, OsDFR, OsPa, OsPs	Meng et al. (2021); Haghi et al. (2022); Galland et al. (2014); Sun et al. (2018)
10	Apiculus	In OsC1-dependent regulation pathways, anthocyanin accumulation is highly dependent on OsC1 and its downstream genes. Accumulation occurs in the apiculi because of the gene interplay amongst OsC1, OsDFR, OsPa, and OsPs. Intensity varies with allelic variations of OsC1	OsC1, OsDFR, OsPa, OsPs	Xia et al. (2021); Meng et al. (2021); Galland et al. (2014)
11	Roots	Limited anthocyanin synthesis due to low expression of major pathway genes	–	Haghi et al. (2022)

## Degradation and Instability of Anthocyanins in Pigmented Rice

**Fig. 5:**
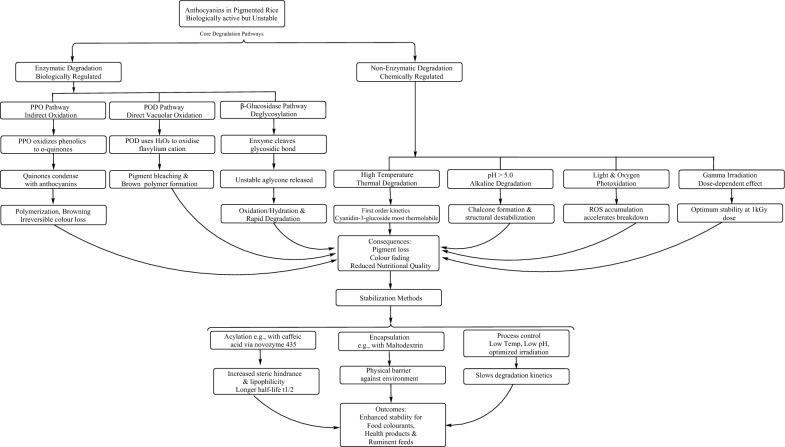
Degradation pathway of anthocyanin

Anthocyanin instability in pigmented rice results from both enzymatic and non-enzymatic degradation processes, which are increasingly recognized as regulated biological phenomena rather than simple chemical breakdown. Environmental factors such as light, temperature, and storage affect anthocyanin accumulation and stability. They are biologically active but unstable anthocyanins. Anthocyanins have a can be degraded very quickly by other factors like pH, light, thermal treatment, enzymes, oxygen, and so on, which limits their overall use (Xia et al. 2021). Consequently, the process of pigmented rice anthocyanin degradation is a result of the synchronized interplay between enzyme activity and environmental conditions, influencing the stability of pigments during the different phases of development, storage, and processing (Fig. 5).

### Enzymatic Degradation of Anthocyanins

Among the mechanisms of pigment turnover in pigmented rice, enzymatic degradation is the main and biotic regulated route. In this process, which is controlled by spatial and temporal factors, the changes of tissues and organs vary depending on the developmental stage, the aging process, and environmental stress responses. In rice, the enzymes responsible for anthocyanin degradation have different expression levels in the bran, pericarp, hull, and vegetative tissues, which leads to the occurrence of colour stability and colour loss specific to the organs (Oren-Shamir 2009; Zhao et al. 2021).

#### Polyphenol Oxidase (PPO) Mediated Oxidative Pathway

Polyphenol oxidase (PPO) is an enzyme that catalyzes the conversion of the native phenolic compounds into the corresponding o-quinones via an oxygen-dependent process. The oxidized forms of the phenolics are very reactive and can rapidly react with non-enzymatic processes to form anthocyanins, which results in the production of pigment polymers, browning, and irreversible colour loss (Oren-Shamir 2009). PPO activity under normal conditions is restricted to plastids but when cells are ruptured due to mechanical injury, milling, senescence, or post-harvest handling the enzyme is free to interact with anthocyanins present in vacuoles in the presence of oxygen. The interaction of PPO with pigments during storage and processing is the main cause of rapid colour loss in pigmented rice bran (Oren-Shamir 2009; Liu et al. 2018).

#### Peroxidase (POD) Driven Vacuolar Oxidation Pathway

Class III peroxidases (PODs) cause direct oxidation of anthocyanins through their use of hydrogen peroxide (H₂O₂) to transform anthocyanins' flavylium cation structure. The reaction proceeds mainly through vacuoles and cell walls which lead to quick pigment disappearance and production of brown polymers (Oren-Shamir 2009; Movahed et al. 2016). The POD enzyme exhibits increased activity when organisms experience oxidative stress or high temperatures or sunlight or when they enter a state of senescence which leads to increased reactive oxygen species (ROS) production. Pigmented rice and fruits and ornamental flowers show increased POD expression and activity which correlates with anthocyanin degradation because POD-mediated degradation functions as a controlled metabolic pathway instead of an automatic chemical process (Movahed et al. 2016; Zhao et al. 2021).

#### β-Glucosidase Induced Deglycosylation Pathway

β-Glucosidases hydrolyzes the glycosidic bond which connects the anthocyanidin aglycone with its sugar moiety to cause anthocyanin degradation. The stability and solubility of anthocyanins depend on their glycosylation which enables their vacuolar sequestration, while their deglycosylation results in unstable anthocyanidins that proceed to undergo oxidation and hydration and structural rearrangement (Oren-Shamir 2009). The activity of β-Glucosidase develops major significance during three specific times which include post-harvest storage and tissue senescence and seed aging because these conditions lead to decreased membrane integrity and loss of compartmental barriers that separate enzymes from anthocyanins. The pathway leads to rapid degradation of pigments because it triggers oxidative reactions that follow it begins (Liu et al. 2018; Zhao et al. 2021).

#### Integrated Enzymatic Degradation Network

The study shows that pigmented rice anthocyanins undergo degradation through an enzymatic network which operates in three distinct processes (i) PPO-generated quinones initiate indirect oxidation while (ii) β-glucosidase enzymes perform deglycosylation to oxidize unstable aglycones and (iii) POD enzymes execute direct oxidation through their action in vacuoles and cell walls (Oren-Shamir 2009).

These pathways frequently operate synergistically under environmental stresses such as high temperature, light exposure, oxidative stress, and nutrient imbalance. Coordinated upregulation of PPO, PODs, and β-glucosidases reinforces the view that anthocyanin degradation is stress-responsive and developmentally programmed, rather than accidental. Research has shown that fruit degradation processes in litchi and grape plants and ornamental flowers use similar enzymatic control systems which demonstrate that these degradation processes remain unchanged across different plant types (Zhao et al. 2021; Machado et al. 2023).

### Non-enzymatic Degradation Pathways

Non-enzymatic degradation pathways are strongly influenced by pH, temperature, light exposure, oxygen availability, and oxidative stress. The rate of anthocyanin degradation occurs with the exposure to light, oxygen, and heat. Anthocyanins are most stable under acidic conditions, whereas alkaline pH, high temperature, and light promote structural destabilization and rapid degradation. Reactive oxygen species (ROS) specifically hydrogen peroxide build up during stress situations which lead to anthocyanin degradation that occurs in vacuoles lacking antioxidant enzyme systems thus establishing another connection between oxidative stress and pigment instability (Zhao et al. 2021).

#### Thermal Degradation

Degradation mechanisms of anthocyanins in black rice bran occur by first-order kinetics, wherein the rate constant increases with the rise in temperature from 60 °C to 100 °C; as such, it is inferred that a higher temperature favours degradation (Loypimai et al. 2016). The longer half-life (t1/2) thus indicates that the irradiated samples manifest higher stability against anthocyanins than the non-irradiated controls. In this endothermic degradation process, the enthalpy is positive (ΔH#), the reaction is non-spontaneous with positive Gibbs free energy (ΔG#), and the entropy is negative, implying a reduction in molecular freedom (ΔS#) (Ito et al. 2019). Furthermore, the intermolecular co-pigmentation resulting from the mixing of various compounds, like sugars and derivatives thereof, also serves to stabilize anthocyanins, but high temperature and sugars facilitate degradation of anthocyanins via the Maillard reaction. Cyanidin-3-glucoside is more thermolabile than the other anthocyanins (Hou et al. 2013). In grapes and other fruits, high temperature stress has been shown to accelerate anthocyanin catabolism, often accompanied by peroxidase activity, suggesting enzymatic as well as chemical degradation (Oren-Shamir 2009).

#### pH-Based Degradation

Anthocyanins experience degradation at low pH levels which stay close to 2.0 and this condition provides them with greater stability because their activation energy (Ea) values at this level. Anthocyanins break down at pH values above 5.0 because their degradation rate increases which results in shorter half-life times that indicate decreased stability at that pH. The activation energy (Ea) shows a decrease with rising pH levels while cyanidin-3-rutinoside exhibits its highest stability at lower pH levels (Hou et al. 2013). This is similarity explorable within Plants, which illustrates the fact that vacuolar pH shifts in plants stress and senescence result in decreased anthocyanin stability and enhanced pigment degradation (Oren-Shamir 2009).

#### Effects of Gamma Irradiation and Stabilization

Gamma-ray doses (0, 1, 2, and 3 kGy) upon anthocyanin stability and thermal degradation kinetics during storage (0, 30, 60, 90, and 120 days) at diverse temperature levels (4, 25, 35, and 45 °C) tend to degrade anthocyanins faster with an increase in temperature. The anthocyanin degradation rate increases with rising temperatures which scientists studied through gamma-ray doses of 0 1 2 and 3 kGy and thermal degradation experiments that lasted 0 30 60 90 and 120 days at multiple temperature settings of 4 25 35 and 45 °C. The maximum anthocyanin stability exists at 1 kGy dose rate while the compound degrades at slower rates when both pH and temperature decrease. Heat at 45 °C eliminates anthocyanins through oxidation while gamma radiation maintains their structural integrity according to (Ito et al. 2019). The process of co-pigmentation and encapsulation creates changes in the stability maintenance of anthocyanins. The maltodextrin used to create powder products protects anthocyanin compounds from environmental exposure while maintaining their visual appearance and nutritional value. The maltodextrin encapsulation method increases anthocyanin stability through the formation of stable complexes which prevent the degradation process of anthocyanins (Loypimai et al. 2016).

#### Organ-Specific Anthocyanin Degradation Patterns

Cyanidin-3-O-glucoside displayed maximum stability because its half-life duration decreased with rising pH levels which made it more enduring than all other anthocyanins. Alkaline conditions (pH > 5.0) lead to fast anthocyanin degradation which results in even quicker half-life reductions. Maltodextrin encapsulation creates stable complexes which protect anthocyanins from degradation thus increasing their stability (Loypimai et al. 2016). The 1 kGy gamma dose showed effective TAC retention throughout all storage temperatures but especially performed well at 4 °C where anthocyanin loss reached only 9.33%. Non-irradiated samples (0 kGy) showed greater anthocyanin reduction at all temperatures because their structural integrity became weakened during higher temperature exposure (Ito et al. 2019). Such organ-specific stability is also observed in other species, for example, leaves where anthocyanins degrade as tissues mature, or flowers like *Brunfelsia*, where pigments are actively degraded post-anthesis via peroxidases and β-glucosidases (Oren-Shamir 2009).

#### Degradation of Visual Colour

As far as the colour is concerned, changes in colour intensity were due to degradation of anthocyanins following the same kinetics. Since the C* value denotes chroma and hence it correlates well with anthocyanin concentration, one may say that visual-colour parameters can be used to monitor anthocyanin stability in real time (Loypimai et al. 2016). Comparable patterns have been reported in ornamental flowers where fading colouration is directly linked with active anthocyanin breakdown rather than dilution alone (Oren-Shamir 2009).

#### In Situ Degradability of Anthocyanins and Proanthocyanidins

Depending on degradability characteristics, the crops are ranked as wheat, barley, rice, and maize. Under in situ conditions, coloured rice grains degrade just like non-pigmented rice with different c and ED values. Starch degradability depends on starch composition (amylopectin/amylose), starch-protein interaction, and granule shape. The variability of c and ED among non-pigmented rice may originate from differences in granule structure, which might also be the explanation of how coloured rice differs in these terms. Grinding with a 2-mm screen would increase starch digestion; most of the DM and starch in rice (a + b) are ruminally decomposed, whereas almost 100% degradability is attained within 48 h, and pigments are released to ruminal fluid, thus making coloured rice the perfect pigment source for ruminants (Hosoda et al. 2018).

## Enhancement of Anthocyanin Stability in Pigmented Rice

Acylation at the hydroxyl sites of the glucosides of black rice anthocyanins and their acyl derivatives is confirmed by UV, FTIR, and UHPLC-HR-MS/MS analyses. Acylation with caffeic acid increases their lipophilicity. Their antioxidant activity in aqueous media drops (traced by ABTS assay), while their DPPH clearance rate and total antioxidant capacity go up post-acylation. These derivatives, however, face a decrease in degradation (value of rate constant k), increased half-life (t1/2), and thus better stability, relative to unacylated ones, under temperature, pH, and light conditions. The enzymatic acylation of anthocyanins extracted from black rice is investigated using Novozyme 435 for the interrelationship of structural modifications with stability and antioxidant properties. Basically, the acylation process takes place at either the C3 and C5 or the C3 and C6 positions on the sugar moiety. The increased stability of acylated derivatives may be largely attributed to their unique steric hindrance, as it constitutes a serious barrier for water to invade the compound. Therefore, the achieved results can open new areas of application of acylated black rice anthocyanins for colouring food and health products as well as in new cereal-based formulations (Xia et al. 2021; Kong et al. 2023).

### Knowledge Gap

Although anthocyanin biosynthesis in pigmented rice has been extensively studied, the genetic regulation of anthocyanin stability and degradation remains poorly understood. Current knowledge gaps can be structured into three hierarchical levels.

(i) **Characterized in rice:** Only a limited set of anthocyanin‐degrading enzymes polyphenol oxidases (PPOs), class III peroxidases (PODs), and β-glucosidases have been indirectly associated with anthocyanin loss in rice tissues during senescence, storage, and stress. While their enzymatic activities correlate with pigment degradation, direct genetic validation of their specific roles in rice anthocyanin stability is still scarce. Similarly, anthocyanin transporters (e.g., GSTs, ABC/MATE proteins) are known to control pigment accumulation, but their contribution to long-term pigment stability has not been systematically evaluated.
(ii) **To be validated:** In the case of model plants and horticultural ones that include *Arabidopsis thaliana*, maize, grape, and litchi, it has been proved that peroxidases, β-glucosidases, and senescence-associated regulators directly mediate the degradation of anthocyanins. Even though the rice genome has homologous genes, their participation in the rice anthocyanin degradation has not been tested yet, which means that there is a significant translational gap.
(iii) **Unexplored:** In rice, the principal regulatory layers have not been investigated, including transcriptional control of degradation enzymes, hormone and ROS-mediated signaling, post-translational regulation, and epigenetic mechanisms. Furthermore, it is still uncertain whether MYB-bHLH-WD40 regulators are not only involved in the biosynthesis but also in the degradation of anthocyanin, which is a major factor in the process.

## Conclusion

Pigmented rice, such as black, red, and purple varieties, is considered nutritionally superior to white rice because of the high content of anthocyanins, proanthocyanidins, and other flavonoids that are mainly present in the bran and pericarp. The bioactive compounds possess very strong antioxidant activity and bring the whole range of health benefits which include anti-diabetic, anticancer, anti-inflammatory, antioxidant, cardioprotective, neuroprotective, and anti-aging effects. Along with the benefits to human health, these pigments also play an important role in ecology as they provide rice plants with the ability to resist the environmental stresses such as UV radiation, oxidative stress, temperature extremes, drought, and so on. The degree of colouring of rice grains is genetically determined primarily through the MYB-bHLH-WD40 (MBW) transcriptional complex; here, the genes OsC1, Kala4, and OsTTG1 can be regarded as the key regulators that activate the structural genes OsDFR and OsANS, hence controlling the tissue-specific production of anthocyanins.

The colouration of red rice is mostly determined by the Rc gene, which regulates flavonoids towards the activation of proanthocyanidins. The pathways of anthocyanins and proanthocyanidins however, have common phenylpropanoid precursors but split at the leucoanthocyanidin stage resulting in different colours. Besides these, gene duplication, allelic variation, epigenetic regulation, and environmental factors are also among the causes of rice pigmentation diversity.

While anthocyanin biosynthesis, transport, and vacuolar storage are relatively well understood, pigment stability remains a major challenge. Anthocyanins are highly unstable and undergo degradation during development, storage, and processing through both enzymatic and non-enzymatic mechanisms. Enzymes that are involved in oxidation and deglycosylation, polyphenol oxidases, peroxidases, and β-glucosidases stand out as the most active ones, while on the other hand, heat, light, oxygen, and alkaline pH are the factors that promote non-enzymatic degradation. In contrast, proanthocyanidins exhibit greater stability due to their condensed polymeric structure, although the regulation of their polymer length, linkage type, and tissue-specific accumulation is still poorly understood.

New methods like acylation, encapsulation, co-pigmentation, and gamma irradiation have been recognised for their potential to enhance anthocyanin's stability, but their genetic and molecular aspects have not been sufficiently investigated yet. Overall, coloured rice is an important source of nutrition, plant survival and genetic variability. Research on revealing the pathways of degradation of anthocyanins, mechanisms of proanthocyanidins polymerization and regulation of their stability needs to be done, while aiming at providing high-yielding, stress-tolerant rice varieties with stable health-promoting pigments through the combination of genomics, gene-editing, and post-harvest technologies.

## Data Availability

No datasets were generated or analysed during the current study.

## Abbreviations

ABC transporters: ATP-binding cassette transporters
ABTS: 2,2'-Azino-bis (3-ethylbenzothiazoline-6-sulfonic acid)
ANS: Anthocyanidin synthase
bHLH: Basic helix-loop-helix
C3G: Cyanidin-3-glucoside
C4H: Cinnamate 4-hydroxylase
CHI: Chalcone isomerase
CHS: Chalcone synthase
CRISPR/Cas9: Clustered regularly interspaced short palindromic repeats/CRISPR-associated protein 9
DAF: Days after flowering
DFR: Dihydroflavonol reductase
DM: Dry matter
DNA: Deoxyribonucleic acid
DPPH: 2,2-Diphenyl-1-picrylhydrazyl
ED: Effective degradability
Ea: Activation energy
ER: Endoplasmic reticulum
F3H: Flavanone 3-hydroxylase
F3′H: Flavonoid 3′-hydroxylase
F3′5′H: Flavonoid 3′,5′-hydroxylase
FTIR: Fourier-transform infrared spectroscopy
GST: Glutathione-S-transferase
GWAS: Genome-wide association study
H_2_O_2_: Hydrogen peroxide
iTRAQ: Isobaric tags for relative and absolute quantitation
kGy: Kilogray (unit of radiation)
LAR: Leucoanthocyanidin reductase
LDL: Low-density lipoprotein
LT: Ligandin transport
MBW complex: MYB-bHLH-WD40 complex
PAL: Phenylalanine ammonia-lyase
PPO: Polyphenol oxidase
POD: Peroxidase
ROS: Reactive oxygen species
SNPs: Single nucleotide polymorphisms
TAC: Total anthocyanin content
UFGT: UDP-glucose flavonoid 3-O-glucosyltransferase
UHPLC-HR-MS/MS: Ultra-high performance liquid chromatography-high resolution tandem mass spectrometry
UV: Ultraviolet
VT: Vesicular Transport
4CL: 4-Coumarate-CoA ligase
ΔG#: Change in Gibbs free energy of activation
ΔH#: Change in enthalpy of activation
ΔS#: Change in entropy of activation
